# Microscopic prevalence and risk factors of asymptomatic malaria in Gorgora, western Dembia, Northwest Ethiopia: exploring hidden threats during minor transmission season

**DOI:** 10.1186/s12936-024-05178-5

**Published:** 2024-12-18

**Authors:** Tena Cherkos, Adane Derso, Wossenseged Lemma, Aberham Abere, Teshiwal Deress, Banchamlak Tegegne, Gebeyaw Getnet Mekonnen, Abebe Birhanu, Yalewayker Tegegne

**Affiliations:** 1 Department of Medical Parasitology, School of Biomedical and Laboratory Sciences, College of Medicine and Health Sciences, University of Gondar, Gondar, Ethiopia; 2 Department of Quality Assurance and Laboratory Management, School of Biomedical and Laboratory Sciences, College of Medicine and Health Sciences, University of Gondar, Gondar, Ethiopia; 3 Amhara Public Health Institute, Bahir Dar, Ethiopia; 4 Department of Medical Microbiology, School of Biomedical and Laboratory Sciences, College of Medicine and Health Sciences, University of Gondar, Gondar, Ethiopia

**Keywords:** Asymptomatic malaria, Associated factors, Gorgora, Prevalence

## Abstract

**Background:**

Malaria poses a significant public health threat globally, particularly in African regions, where asymptomatic malaria is a considerable logistic problem. Individuals with asymptomatic malaria do not seek treatment, and thus they are invisible to health facilities and represent a substantial hidden reservoir of *Plasmodium* species. This study aimed to determine the prevalence of asymptomatic malaria and its associated factors in Gorgora, western Dembia district, Northwest Ethiopia.

**Methods:**

A community-based cross-sectional study was conducted from May to June 2023 in the Gorgora area, Western Dembia district, Northwest Ethiopia. Data were collected using a semi-structured questionnaire. Giemsa-stained blood smear microscopy was employed for the diagnosis of *Plasmodium* species. The data were entered into Epi Data version 4.6 and exported to SPSS version 25 for analysis. Bivariate and multivariable binary logistic regression analyses were conducted to identify associated factors.

**Results:**

Among the 357 individuals who participated in this study, 9.2% (33/357) [95% CI 6.40–12.70: p = 0.000] were confirmed to be infected with *Plasmodium* species. *Plasmodium falciparum* and *Plasmodium vivax* accounted for 66.7% and 33.3%, respectively. Not using bed nets [AOR = 7.3, 95% CI 2.08–23.46, p = 0.006)], previous malaria history [AOR = 2.6, 95% CI 1.01–6.45, p = 0.041], outdoor activities at night [AOR = 8.3, 95% CI 3.21–21.30, p = 0.000], and family size [AOR = 3.3, 95% CI 1.18–9.22, p = 0.023] were significantly associated with asymptomatic malaria (p < 0.05).

**Conclusions:**

A considerable proportion of asymptomatic *Plasmodium* infections was found which likely act as a reservoir of transmission. This has implications for ongoing malaria control programmes that are based on the treatment of symptomatic patients and highlight the need for intervention strategies targeting asymptomatic carriers. Not using bed nets, engaging in outdoor activities at night, and having a family size of more than five increased the odds of developing asymptomatic malaria. The district health office and health extension workers should collaborate to promote the regular use of mosquito bed nets among community residents.

## Background

Malaria poses a substantial public health threat on a global scale [1–4]. In 2022, there were an estimated 249 million malaria cases and 608,000 deaths, a concerning increase of 5 million cases compared with 2021 [5]. The disease is caused by species of *Plasmodium*, with *Plasmodium falciparum* and *Plasmodium vivax* being the most prevalent species in Ethiopia, contributing 60%–70% and 30%–40% of malaria cases, respectively [6]. In Ethiopia, approximately 75% of the landmass is considered malarious, with 68% of the total population living in areas at risk of malaria [7–9]. The main malaria vectors in the country are *Anopheles arabiensis* followed by *Anopheles pharoensis* [9]. Malaria prevalence in Ethiopia is seasonally influenced with peaks coinciding with the planting and harvesting seasons, posing an economic challenge. This results in an economic burden, which can adversely affect the fight against poverty [10].

The clinical manifestation of malaria ranges from asymptomatic to severe and complicated infections [11]. The likelihood of contracting asymptomatic malaria can be increased by factors like living conditions and housing structures, as these can promote mosquito vector entry, indoor resting, biting, and ultimately malaria transmission [12]. Repeated exposures to *Plasmodium* infections can result in the development of partial immunity, offering some protection against further complications [13]. Asymptomatic malaria is the presence of *Plasmodium* parasites in the blood without fever and other acute malaria symptoms in individuals who have not received recent antimalarial treatment [14, 15]. Furthermore, malaria endemicity and other factors such as age, bed net utilization, genetic background, and the level of partial immunity from previous exposures can all influence the frequency of asymptomatic malaria [16].

Stable endemic regions and areas with unstable transmission have a significant prevalence of asymptomatic malaria carriers [17–19]. Most carriers are unaware of the infection and do not seek treatment, and, therefore, remain invisible to the healthcare system [20, 21]. Parasites from asymptomatic carriers are infectious to *Anopheles* mosquitoes, and their continuous exposure to mosquito bites fuels the transmission cycle [13, 20, 22]. Asymptomatic *Plasmodium* species carriers can pose a serious threat to malaria control and elimination efforts [21, 23]. Beyond sustaining transmission, asymptomatic malaria has been linked to adverse health effects, including anaemia among pregnant women and children, as well as chronic malnutrition and cognitive impairment in children [21]. Individuals with asymptomatic malaria may also develop symptomatic malaria at a later stage [24, 25]. In Ethiopia, studies have reported a high prevalence of asymptomatic infections [8, 26, 27].

Intending to eliminate malaria nationwide by 2030, the Federal Ministry of Health has launched a sub-national malaria elimination initiative in 2017 [28]. A successful malaria elimination programme calls for attention to all parasite carriers, including asymptomatic malaria cases [29].

Most epidemiological studies of malaria in Ethiopia have focused on symptomatic malaria. Studies on asymptomatic malaria have been limited, with a primary emphasis on pregnant women and children [18, 30]. Limited data on asymptomatic malaria parasite reservoir potentially under-estimates the malaria burden, undermines efforts for parasite clearance, and compromises opportunities for transmission interruption and subsequent efforts to achieve malaria elimination. Therefore, this study aimed to assess the prevalence and associated factors of asymptomatic malaria among community residents in the study area.

## Methods

### Study area and period

**Fig. 1:**
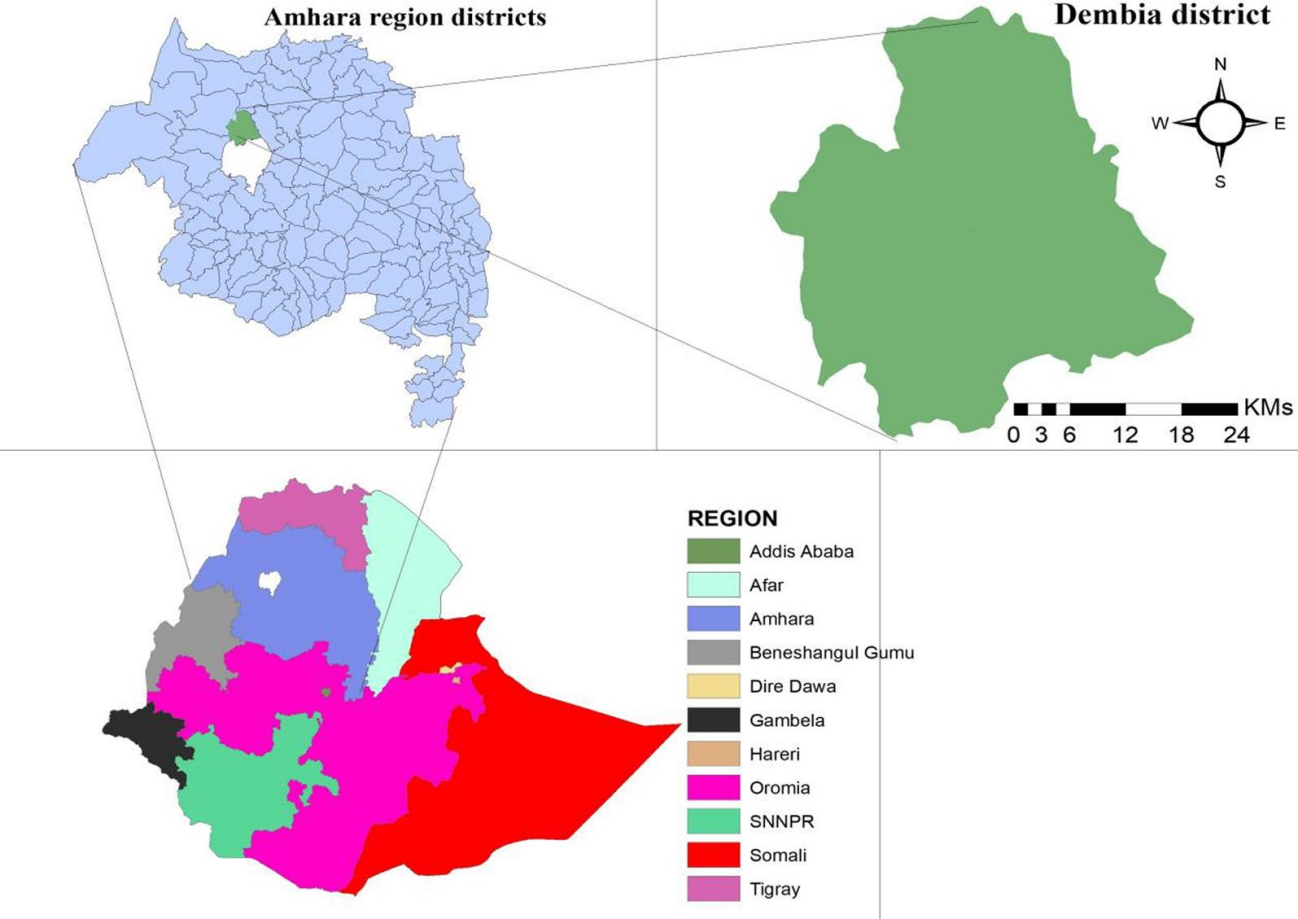
Map of the study site at Western Dembia District, Northwest Ethiopia. N: number of households in the Kebele. n: selected household in the kebele

A community-based cross-sectional study was conducted from May to June 2023 in Gorgora, western Dembia district, Northwest Ethiopia to assess the prevalence of asymptomatic malaria and identify associated factors among community dwellers. Gorgora is situated 65 kms from Gondar town and 808 kms from Addis Ababa, the capital city of Ethiopia [31]. The climate in Gorgora can be described as moist Weina Dega, with rainfall ranging between 900 and 1400 mm, and the altitude falling within the range of 1500 to 2300 m above sea level. The temperature in Gorgora varies from 24 to 15 °C [31]. Its geographical coordinates are 12° 14ʹ N latitude and 37° 18ʹ E longitude. The area has an estimated total population size of 16,270 [31]. The local economy primarily depends on trading, farming, and fishing. Gorgora is characterized by ongoing malaria transmission, with an estimated prevalence rate of 30.3%. The most prevalent *Plasmodium* species in the area are *P. falciparum* and *P. vivax* [7] (Fig. 1).

### Population and inclusion criteria

The study population for this research included all individuals who were residents of Gorgora, Western Dembia district, Northwest Ethiopia. In each selected kebele, individuals from the selected households were included in the study if they met the following criteria. Firstly, individuals had to be permanent residents of the area, and they had to have resided in the area for a minimum of 6 months or longer. Secondly, individuals were required to show no signs or symptoms of malaria and have an axillary temperature below 37.5 °C. They also should not have had a history of fever within the past 72 h and should not have taken any anti-malarial treatment for at least one month before and during the data collection period. Moreover, individuals were required to provide informed written consent to participate in the study, and for children below 18 years written informed consent should be taken from their parents/guardians. On the other hand, individuals who were severely ill, unable to respond, or unable to provide sufficient blood samples for various reasons were excluded from the study.

### Sample size and sampling technique

The required sample size was calculated using the single-population proportion formula considering a 95% confidence interval (CI), a design effect of 2, a margin of error of 5%, and based on a 12% asymptomatic malaria prevalence reported in a prior study [8]. By adding a 10% non-response rate, the final sample size was calculated as follows:

$${\text{n}} = \left( {{\text{Z}}\upalpha /{2}} \right){\text{2P }}\left( {{1} - {\text{P}}} \right) \times {\text{DeEf}}/{\text{d2}})$$

$${\text{n}} = \left( {{1}.{96}} \right){2} \times \left( {0.{12}} \right) \times \left( {0.{88}} \right)/\left( {0.0{5}} \right){2} \times {2}$$

$${\text{n}} = {324} + \left( {{324} \times 0.{1}} \right)$$

$${\text{n}} = {357}$$

where n is the required sample size. Zα/2 is the value under the standard normal table for the given value of confidence level = 1.96. P is the prevalence of asymptomatic malaria from a previous study conducted at Gondar Zuria district. d is the margin of error. DeEf is the design effect.

**Fig. 2:**
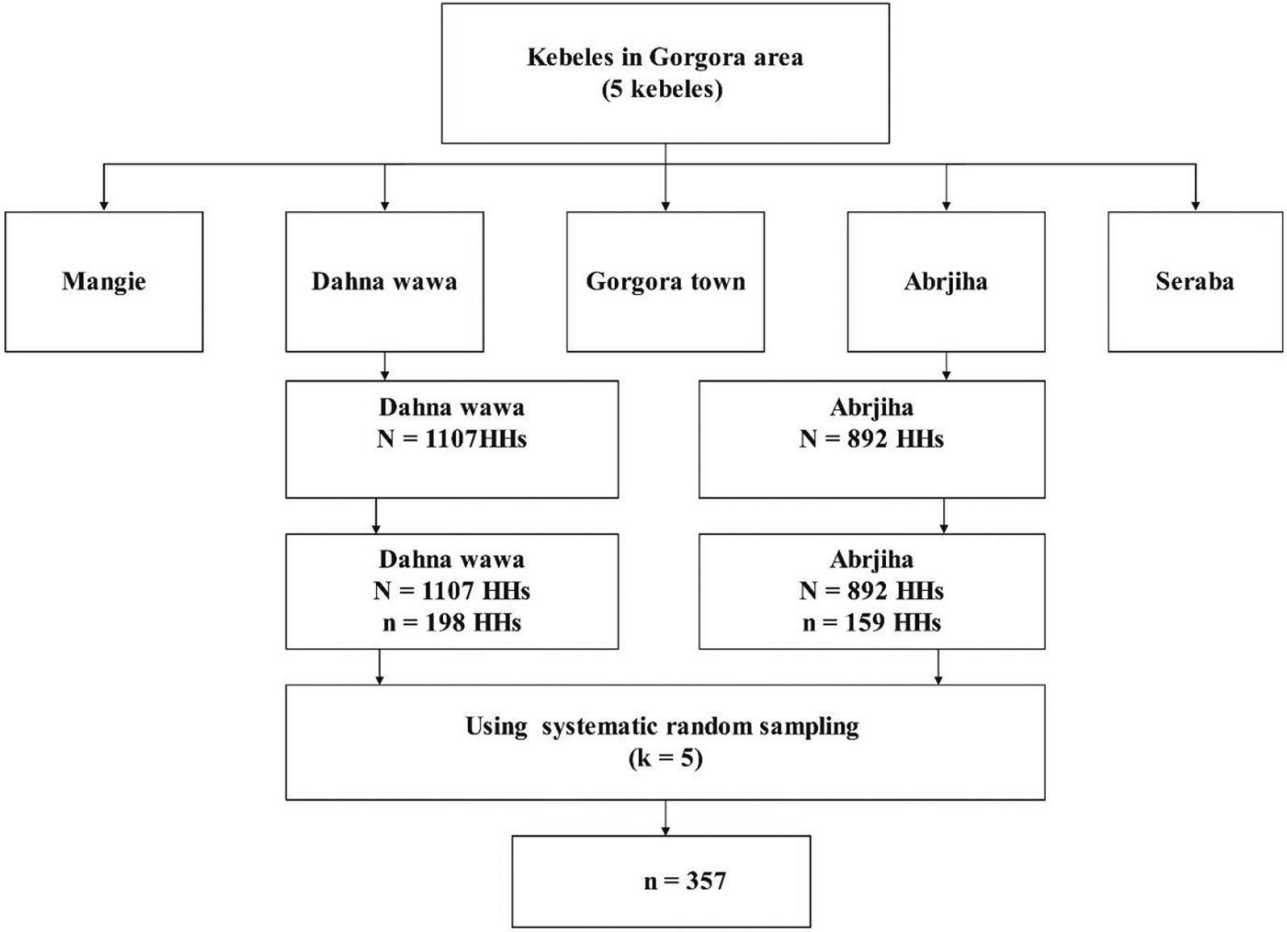
Schematic presentation of sampling procedure

A multi-stage sampling technique was used to select the study participants. In the first stage, two kebeles (Danawawa and Abrjiha) were selected using the lottery method. At the second sampling stage, the number of households (HHs) in each kebele was determined using proportional allocation. Systematic random sampling was then used to select HHs with an interval of five. The sampling interval was calculated by dividing the total number of HHs by the number of HHs to be included in the sample from each kebele. The initial HH was randomly selected by lottery method and the next HH was selected at that interval. One study participant was selected from each HH regardless of family size using a lottery method until 357 individuals were sampled. In case no eligible participant was identified in a selected HH, the next HH was selected keeping the interval constant afterward (Fig. 2).

### Dependent and independent variables of the study

The dependent variable of the study was the prevalence of asymptomatic malaria. The independent variables included age, sex, family size, occupational status, educational status, presence of holes in the house wall, presence of a kitchen in the main house with no partition, previous history of malaria, family history of malaria, outdoor activities at night, bed net usage, and presence of stagnant water.

### Operational definitions of asymptomatic malaria

The presence of *Plasmodium* parasites in the blood of a person with no history of symptoms and /or signs of malaria within the past 2 days and at the time of the survey, whose body temperature is < 37.5 °C at presentation and no history of fever for the past 72 h [8, 18].

#### Questionnaire data

The questionnaire, which includes socio-demographics and other variables of the study, was developed in the English language after reviewing previous literature and translated into the local language (Amharic). The questionnaire was pre-tested with 5% of the total sample size among community residents outside of the selected kebeles three weeks before actual data collection time, and necessary modifications were made based on pretest findings. For instance, the variable “sometimes” in bed net utilization and “merchant” in occupation were added. Before starting actual data collection, training was given to data collectors regarding the objective of the study, study participant recruitment techniques among household members, the data collection instrument, data collection techniques, and other ethical issues by the principal investigator of this study. Subsequently, trained questionnaire administrators conducted face-to-face interviews with respondents in the participant house by reading the questionnaires, alongside capillary blood sample collection.

#### Blood sample collection and laboratory diagnosis

Before capillary blood sample collection, the inclusion criteria used to enroll the study participants were screened by a nurse professional with a qualification of BSc degree. After obtaining written informed consent from study participants who fulfilled the inclusion criteria, capillary blood samples were collected aseptically from finger pricks, using sterile blood lancets by two trained laboratory technologists. The first drop of blood was removed, and consecutive drops were taken for blood film preparation. Thin and thick blood smears were prepared on a single slide for each of the study participants by dropping approximately 2–3 μl and 6 μl blood respectively. Each blood smear was air-dried, and the thin smear was fixed by carefully dropping methanol using a Pasteur pipette. The methanol-fixed thin smears were allowed to dry completely in the air by placing the slides on a flat surface. Then, the dried blood smears were transported to the nearby health centre in a slide box to stain with 10% Giemsa for 10 min. Finally, microscopic slides were immediately transported to and examined under oil emulsion (100 X) objective at the University of Gondar medical parasitology laboratory to detect asymptomatic malaria and identify *Plasmodium* species using thick and thin blood smears respectively. A slide was considered negative if no *Plasmodium* parasite was detected after examination of at least a hundred fields of the thick smear with a 100X objective [32]. All microscopic slides were examined independently by two experienced laboratory technologists. The first and second laboratory technologists who examine malaria microscopic slides have eight and ten years of malaria microscopy experience at the University of Gondar Comprehensive Specialized Hospital respectively. The discordant results were reexamination by a third trained and certified laboratory technologist blind to the initial examination results. A third laboratory technologist who read discordant results has certification during completing several malaria microscopy trainings such as FHI 360 malaria microscopy training, August 11–15, 2014, University of Gondar Hospital, Gondar, Ethiopia, and training on malaria laboratory diagnosis and quality assessment organized by Ethiopian public health institute (EPHI) in collaborated with ICAP—Colombia University programs in Ethiopia and PMI/USAID Ethiopia held on August 03–07, 2015.

### Data processing and analysis

Data were coded, entered into Epidata version 4.6, cleaned up, and analysed using SPSS for Windows version 25 (IBM SPSS Statistics 25). Frequencies and summary statistics, such as mean, standard deviation, and percentages, were generated to describe the study participants in terms of the relevant variables. The Chi-square assumption was checked for all categorical independent variables. Binary logistic regression was used to assess the associations between dependent and independent variables. In the bivariate logistic regression analysis, variables with a p-value < 0.25 were considered potential candidates for the multivariable logistic regression analysis. Variables with a p-value < 0.05 from the multivariable regression analysis were considered statistically significant. The goodness of fit of the model was assessed using the Hosmer–Lemeshow test. Finally, the results of this study were presented in text, tables, and figures accordingly.

### Data quality control and management

Quality control measures were implemented for the working equipment and reagents, using standard controls. The quality of the Giemsa solution was ensured by checking stock expiration dates, filtering the Giemsa working solution, and employing known positive and negative control slides.

## Results

### Socio-demographic characteristics

The study included a total of 357 asymptomatic participants from two kebeles. Of this total, 53.2% (190/357) were females. The mean age of the participants was 26.8 years, with a standard deviation of ± 14.04 years. In terms of educational status, 45.1% (161/357) were reported as illiterate. Additionally, most of the participants, comprising 63.3% (226/357), were engaged in farming (Table 1).

**Table 1:** Socio-demographic characteristics of study participants in Gorgora, Northwest Ethiopia 2023

Variables	Category	Frequency	Percentage
Age	5–14	69	19.3
	15–29	145	40.6
	> 29	143	40.1
Sex	Male	167	46.8
	Female	190	53.2
Family size	≤ 5	166	46.5
	> 5	191	53.5
Occupation	Farmer	226	63.3
	Government employee	29	8.1
	Merchant	35	9.8
	Student	67	18.8
Educational status	Illiterate	161	45.1
	Elementary	140	39.2
	High school and above	56	15.7

### Behavioural, environmental, and other factors of study participants

Over one-fifth (23.2%, 83/357) of participants engaged in outdoor activities at night, placing them at increased risk of mosquito bites. Furthermore, a significant proportion (41.7%, 149/357) did not use bed nets, a key preventative measure against malaria transmission. Additionally, 44.0% (157/357) of participants reported a prior malaria infection, highlighting the prevalence of the disease in the study population. Notably, stagnant water, a breeding ground for mosquitoes, was present near the villages of 40.3% (144/357) of participants (Table 2).

**Table 2:** Behavioural, Environmental, and other factors of study participants in Gorgora, Northwest Ethiopia 2023

Variables	Category	Frequency	Percentage
Outdoor activities at night	Yes	83	23.2
	No	274	76.8
Utilization of bed net	Daily using	92	25.8
	Sometimes	116	32.5
	Not using	149	41.7
Presence of stagnant water	Yes	144	40.3
	No	213	59.7
Presence of holes in the house wall	Yes	150	42.0
	No	207	58.0
Presence of a kitchen in the main house with no partition	Yes	201	56.3
	No	156	43.7
Previous history of malaria	Yes	157	44.0
	No	200	56.0
Family history of malaria	Yes	242	67.8
	No	115	32.2
Kebele	Dana Wawa	198	55.5
	Abrjiha	159	44.5

### Prevalence of asymptomatic malaria

**Fig. 3:**
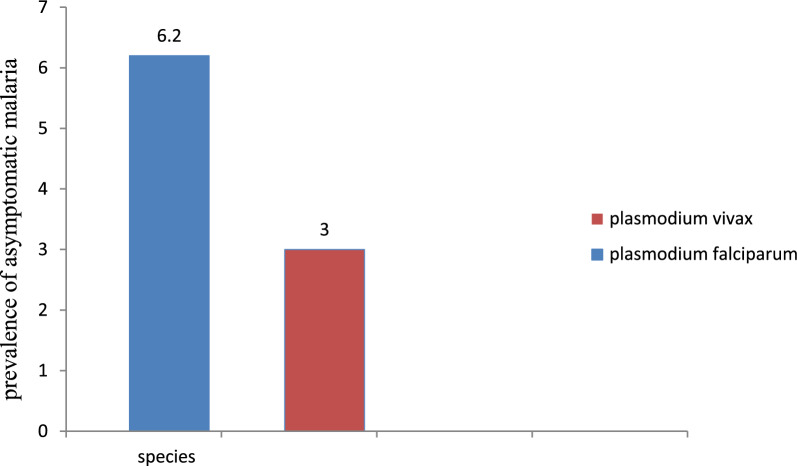
Prevalence of asymptomatic malaria in Gorgora, western Dembia, Northwest Ethiopia

This study found a 9.2% (33/357) prevalence of asymptomatic malaria, with *P. falciparum* and *P. vivax* accounting for 6.2% (22/357) and 3% (11/357) of infections, respectively (Fig. 3).

In the present study, asymptomatic malaria among males and females were 14/357(3.9%) and 19/357 (5.3%) respectively. Similarly, the prevalence of asymptomatic malaria in the age groups of 15–29 and > 29 years were 4.8% (17/357) and 3.3% (12/357) respectively (Table 3).

**Table 3:** Prevalence of asymptomatic malaria by sex and age group in Gorgora, western Dembia, Northwest Ethiopia 2023

	Asymptomatic *Plasmodium* Infection Status				Total	χ^2^	p value
	Pos	Percentage	Neg	Percentage			
Sex							
Male	14	3.9	153	42.9	167	0.599	0.599
Female	19	5.3	171	47.9	190		
Total	33	9.2	324	90.8	357		
Age							
5–14	4	1.1	65	18.2	69	0.339	0.384
15–29	17	4.8	128	35.9	145		
> 29	12	3.3	131	36.7	143		
Total	33	9.2	324	90.8	357		

*Pos* Positive, *Neg* Negative

Notably, 1.1% (4/357) of the total blood samples collected were positive for the gametocyte stage of *P. falciparum.* The prevalence of gametocyte carriage among participants within the age group of 15–29 was 0.8% (3/357) (Table 4).

**Table 4:** Prevalence of gametocyte carriage by sex and age group in Gorgora, western Dembia, Northwest Ethiopia 2023

	Gametocyte status				Total	χ^2^	p value
	Pos	Percentage	Neg	Percentage			
Sex							
Male	1	0.3	166	46.5	167	0.384	0.389
Female	3	0.8	187	52.4	190		
Total	4	1.1	353	98.9	357		
Age							
5–14	0	0	69	19.3	69	0.335	0.639
15–29	3	0.8	142	39.8	145		
> 29	1	0.3	142	39.8	143		
Total	4	1.1	353	98.9	357		

*Pos* Positive, *Neg* Negative

### Factors associated with asymptomatic malaria

From the multivariable regression analysis, bed net utilization (AOR = 7.3, 95% CI 2.08–23.46, p = 0.006), previous history of malaria (AOR = 2.6, 95% CI 1.01–6.45, p = 0.041), outdoor activities at night (AOR = 8.3, 95% CI 3.21–21.30, p = 0.000), and family size (AOR = 3.3, 95% CI 1.18–9.22, p = 0.023) were significantly associated with the prevalence of asymptomatic malaria (p < 0.05). Regarding this finding, respondents who did not use bed nets were 7.3 times (AOR = 7.3, 95% CI 2.08–23.46, p = 0.006) more likely to have asymptomatic malaria than those who use it daily. Study participants who have a previous history of malaria were 2.55 times (AOR = 2.6, 95% CI 1.01–6.45, p = 0.041) more likely to develop asymptomatic malaria than participants who didn’t have it. Engaging in outdoor activities at night was associated with an 8.27-fold increased risk of asymptomatic malaria (AOR = 8.3, 95% CI 3.21–21.30, p = 0.000) compared to those who did not participate in such activities. In addition, participants who had more than five family members were 3.3 times (AOR = 3.3, 95% CI 1.18–9.22, p = 0.023) more likely to have asymptomatic malaria than their counterparts (Table 5).

**Table 5:** Bivariate and multivariable logistic regression analysis of associated factors for asymptomatic malaria in Gorgora, Northwest Ethiopia 2023

Variables	Category	Asymptomatic malaria		OR 95% CI		*p*-value
		Positive	Negative	COR 95%CI	AOR 95% CI	
Age	5–14	4	65	1	1	
	15–29	17	128	2.2 (0.70–6.68)	1.6 (0.41–6.45)	0.492
	> 29	12	131	1.5 (0.46–4.80)	0.7 (0.15–2.86)	0.567
Bednet utilization	Daily	3	89	1	1	
	Sometimes	5	111	1.3 (0.31–5.74)	0.7 (0.14–3.79)	0.709
	No	25	124	6.0 (1.75–16.97)	7.3 (2.09–23.47)	0.006*
Presence of holes in the house wall	Yes	19	131	2.0 (0.97–4.13)	2.4 (0.92–6.15)	0.072
	No	14	193	1	1	
History of malaria	Yes	17	140	1.4 (0.68–2.86)	2.6 (1.01–6.46)	0.041*
	No	16	184	1	1	
Family history of malaria	Yes	29	213	3.8 (1.30–11.02)	2.9 (1.07–8.57)	
	No	4	111	1	1	0.082
Outdoor activities at night	Yes	22	61	8.6 (3.97–18.73)	8.3 (3.22–21.31)	0.000*
	No	11	263	1	1	
Family size	> 5	24	167	2.5 (1.54–5.58)	3.3 (1.18–9.22)	0.023*
	≤ 5	9	157	1	1	

^*^Statistically significant, *COR* Crude Odds Ratio, *AOR* Adjusted Odds Ratio

## Discussion

Light microscopy is the gold standard for the diagnosis of malaria because, when properly interpreted, a positive result indicates an active *Plasmodium* parasite infection and allows evaluation of parasite morphology, and differentiation between *Plasmodium* species [8, 33]. Despite these advantages, light microscopy may not be sensitive enough to be used in cases of low-density parasite carriage [34]. Polymerase chain reaction (PCR), a very sensitive molecular technique, is still the gold standard for diagnosing submicroscopic malaria parasites [35]. However, it is less useful for malaria mass screening programmes in communities with low resources due to the high prices of the sophisticated equipment required, the time-consuming procedure that delays the release of results, and the requirement for skilled laboratory personnel [36].

Identification of *Plasmodium* parasites in asymptomatic individuals is crucial for the reduction of transmission, control, and elimination of malaria. However, due to the low health-seeking behaviuor of asymptomatic individuals, asymptomatic malaria is often undetected and remains untreated [17, 37]. At this time, even with appropriate treatment for symptomatic cases, asymptomatic individuals continue to be potential carriers and transmitters of gametocytes, resulting in ongoing malaria transmission within a population [38, 39]. The present results also indicate that in the study area, treating symptomatic cases alone did not stop or reduce malaria transmission as expected. The high prevalence of asymptomatic malaria obtained by this study, 9.2% could be the source of forward transmission. Thus, addressing the problem associated with asymptomatic carriers is required in the study area or elsewhere to achieve the malaria elimination goals.

The current study found a 9.2% (95% CI 6.40–12.70) prevalence of asymptomatic carriers, consistent with findings from other parts of Ethiopia (10.2% and 12%) [8, 10] and Tanzania (8%) [40]. This prevalence is lower than those reported from other areas of Ethiopia (18.4% and 17.5%) [26, 27], Uganda (34.7%) [41], Ghana (27%) [42], and Nigeria (69.9%) [43]. The current result is higher than the findings of 3.75% and 5% from Ethiopia [44, 45], and Myanmar (1.44%) [46].

These variations in the prevalence of asymptomatic malaria could be attributed to several factors. Seasonality of malaria transmission could be one factor. The present study was conducted in a minor transmission season (May to June), while studies in West Armachiho and Metema district were conducted in the major malaria transmission season (September to December) [26, 27], resulting higher numbers of asymptomatic malaria cases [28].

The study population is another factor. This finding was carried out among the general population, whereas the aforementioned studies in West Armachiho and Metema [26, 27] focused on seasonal migrant workers who had repeated malaria exposures due to frequent visits to malaria-endemic lowland areas. This frequent exposure might allow the development of immunity and allow the parasite to persist in their blood for extended periods without exhibiting signs and symptoms, resulting in a higher prevalence of asymptomatic malaria [47].

This single cross-sectional study design differs from the longitudinal surveys conducted in Ghana [42] which were conducted in forest ecological zones with more favourable conditions for mosquito breeding [47]. Additionally, the Nigerian study [43] targeted household members of symptomatic malaria cases, which can lead to a higher detection rate of asymptomatic carriers compared to the general population approach used in the present study [48].

Seasonality and transmission intensity also influence prevalence. While the present study was conducted during the minor malaria transmission season, the study in North Gondar, Ethiopia [44] was conducted during the dry season with low mosquito breeding activity. Conversely, studies in the West Arsi Zone, Ethiopia [45], and Myanmar [46] were conducted in low transmission settings. In high-transmission areas, individuals frequently exposed to malaria might develop partial immunity that renders *Plasmodium* infection asymptomatic [49], Whereas, in areas of low transmission, populations have limited exposure and thus, are more likely to develop high-density symptomatic parasitaemia.

The present study revealed that the dominant *Plasmodium* species detected was *P. falciparum* (66.7%, 22/33). This finding is consistent with previous studies conducted in different areas of the country [10, 50, 51]. In contrast to the present study, studies conducted in East Shewa [52] and Hadiya Zone [53] reported that *P. vivax* was the dominant *Plasmodium* species over *P. falciparum*. This discrepancy may result from variations in the epidemiological distribution of *Plasmodium* species in various Ethiopian regions, most likely as a result of climate and altitudinal differences [54].

This study revealed that bed net usage, previous history of malaria infection, outdoor activities at night, and family size were significantly associated with the prevalence of asymptomatic malaria (p < 0.05). Respondents who did not use bed nets were 7.3 times more likely to have asymptomatic malaria than those who used bed nets daily. Similar results were reported from studies conducted in Northwest Ethiopia [9, 10, 55] and Uganda [41]. Daily bed net use reduces the risk of malaria infection by preventing human-mosquito contact, which interrupts malaria transmission [18].

Previous history of malaria infection was also a significant factor. Participants with such a history were 2.55 times more likely to have asymptomatic malaria than those who did not. This is consistent with reports from other regions in Ethiopia [8, 26]. The rationale for this finding might be due to the relapsing behavior of *P. vivax* and the recrudescence behavior of *P. falciparum*, where the parasite is likely to be present in the blood after medication [49]. For instance, asymptomatic *P. falciparum* can persist for decades [56]. In addition, encountering multiclonal *P. falciparum* infections might protect against symptomatic malaria and allow the infected individual to remain an asymptomatic carrier [57].

Study participants who were involved in outdoor activities at night were 8.27 times more likely to develop asymptomatic malaria than those who did not. This finding is consistent with studies conducted in Ethiopia [10, 26, 30]. This could be explained by the fact that individuals who are involved in outdoor activities at night are easily exposed to exophagic-exophilic mosquito bites and get *Plasmodium* infections [58].

Furthermore, this study revealed that the odds of developing asymptomatic malaria among participants with more than five family members were 3.3 times higher than those with five or fewer family members. Studies conducted in Ethiopia also showed similar findings [59–61]. Studies carried out in Ethiopia also showed similar findings [59–61]. The reason behind this could be that, as family size increases, there might be individuals who don’t have a chance to use bed nets as universal coverage of mosquito nets is low in the country in general. Moreover, when the number of residents in the HH increases, the olfactory cues to attract the *Anopheles* mosquito become stronger and increase the chance of being bitten by the vector [62].

### Limitations of the study

The study had a few limitations that need to be acknowledged. As a cross-sectional study, it was unable to establish a direct temporal association between asymptomatic malaria and its potentially associated factors. Due to limited resources, the study was unable to utilize molecular tools for detecting asymptomatic malaria, which could have provided additional support to the microscopic investigation. Not using RDTs was another limitation of this study due to a shortage of resources. Therefore, the use of microscopy alone may underestimate the prevalence of asymptomatic malaria. Despite these limitations, the study provides valuable insights into the prevalence and potential factors associated with asymptomatic malaria, albeit without establishing a definitive causal relationship.

## Conclusions

This study reveals a considerable prevalence of asymptomatic malaria. Factors such as the lack of bed net utilization, previous history of malaria, engagement in outdoor activities at night, and larger family size were found to increase the odds of developing asymptomatic malaria. Therefore, the district health office and health extension workers should collaborate to increase the distribution of mosquito bed nets, considering family size, and promote their daily utilization to reduce the prevalence of asymptomatic malaria. Based on these findings, the authors recommend that the responsible authorities focus on eliminating asymptomatic malaria in the study area. Furthermore, in addition to passive case detection, active case detection at the community level could play a crucial role in reducing these silent transmission reservoirs. In addition, further research on the role of asymptomatic carriers in malaria transmission is recommended.

## Data Availability

No datasets were generated or analysed during the current study.

## Abbreviations

CI: Confidence interval
HH: Household
